# Molecules that Inhibit Bacterial Resistance Enzymes

**DOI:** 10.3390/molecules24010043

**Published:** 2018-12-22

**Authors:** Yuan Liu, Ruichao Li, Xia Xiao, Zhiqiang Wang

**Affiliations:** 1 Institute of Comparative Medicine, College of Veterinary Medicine, Yangzhou University, Yangzhou 225009, China; liuyuan2018@yzu.edu.cn (Y.L.); rchl88@yzu.edu.cn (R.L.); xiaoxia@yzu.edu.cn (X.X.); 2 Jiangsu Co-innovation Center for Prevention and Control of Important Animal Infectious Diseases and Zoonoses, Yangzhou 225009, China

**Keywords:** antibiotic resistance, bacterial enzymes, molecules, therapeutic potential

## Abstract

Antibiotic resistance mediated by bacterial enzymes constitutes an unmet clinical challenge for public health, particularly for those currently used antibiotics that are recognized as “last-resort” defense against multidrug-resistant (MDR) bacteria. Inhibitors of resistance enzymes offer an alternative strategy to counter this threat. The combination of inhibitors and antibiotics could effectively prolong the lifespan of clinically relevant antibiotics and minimize the impact and emergence of resistance. In this review, we first provide a brief overview of antibiotic resistance mechanism by bacterial secreted enzymes. Furthermore, we summarize the potential inhibitors that sabotage these resistance pathways and restore the bactericidal activity of inactive antibiotics. Finally, the faced challenges and an outlook for the development of more effective and safer resistance enzyme inhibitors are discussed.

## 1. Introduction

The emergence, prevalence, and rapid spread of antibiotic resistance in pathogens represent an urgent threat to our medical system for the treatment of bacterial infectious diseases [1,2,3]. According to the Centers for Disease Control and Prevention (CDC) reports, at least 2,000,000 illnesses are caused by resistant bacteria annually in the USA that result in approximately 23,000 deaths [4]. There is no doubt that we have entered the post-antibiotic age [5], in which multidrug resistant (MDR) bacteria are increasingly disseminating in the community and cannot be effectively treated by commonly used antibiotics. Worryingly, the sluggish progress in the development of new antibiotics with unique modes of action further exacerbates this problem [6,7]. Although billions of dollars and longer than a decade have been invested, only a few antibiotics were approved by the Food and Drug Administration (FDA) in the past decades [8,9]. Despite these ongoing efforts [10,11,12,13,14,15,16,17,18,19,20], the rigorous challenges faced in novel antibiotics discovery require additional strategies to tackle multidrug-resistant bacteria. Antibiotic adjuvants [21,22], an alternative to antibiotics (i.e., antibodies, probiotics, vaccines, and anti-virulence drugs) [23,24,25], offer two feasible approaches [26]. Given that these alternatives to antibiotics are mainly administered as adjunctive or preventive therapies in a clinical setting [27], antibiotic adjuvants such as resistance enzyme inhibitors are currently the most promising in the fight against antibiotic-resistant bacteria from both scientific and commercial sides.

These compounds that directly inhibit antibiotic resistance could be explored as antibiotic adjuvants. Thus, elucidation of antibiotic resistance mechanisms would contribute to novel antibiotic adjuvants’ discovery. To date, the molecular mechanisms of antibiotic resistance involve reduced cell membrane permeability, chemical structure changes of targets, active efflux of the intracellular antibiotics, and inactivation of antibiotics by resistance enzymes [5,28,29]. Taking into account these resistance mechanisms, the inhibitors of inactivating enzymes and efflux pumps have great promise as antibiotics’ adjuvants. Theretofore, small molecule inhibitors that target the bacterial efflux pumps have been previously reported [30,31,32,33,34] and systematically reviewed [35,36,37]. Notably, the antibiotic resistance mediated by bacterial enzymes poses an unmet clinical challenge, particularly for those antibiotics that are recognized as “last-resort” defense. For example, metallo-β-lactamases (MBLs) such as New Delhi metallo-β-lactamase 1 (NDM-1) [38] could hydrolyze the carbapenems, and plasmid-mediated colistin resistance (mediated by MCR-1) [39] could decrease the affinity between colistin and lipopolysaccharide (LPS) of the outer membrane in Gram-negative bacteria [40]. Therefore, inhibition of resistance enzyme activity provides a potential therapeutic approach for fighting antibiotic-resistant bacteria.

Different from conventional antibiotics that directly target the components or progress that are essential for bacterial growth, inhibitors of resistance enzyme always have no or little direct antibacterial activity. However, these inhibitors could decrease the activity of resistance enzymes by specifically binding to the active site or inhibiting the transcription and translation of resistance genes. For example, clavulanic acid, a β-lactam-containing natural product, is a valuable inhibitor of β-lactamase [41]. Most notably, enzyme inhibitors may have the significant advantage that they do not exert as much selective pressure as conventional antibacterial agents. They do not directly target growth or viability, thereby conceivably minimizing the evolution of resistance [21]. This review offers a brief overview of antibiotic resistance mechanisms promoted by bacterial secreted enzymes and the development of potent resistance enzyme inhibitors in the battle against antibiotic-resistant bacteria. Besides, challenges and outlook for future efforts to discover therapeutic resistance inhibitors are also discussed.

## 2. Antibiotic Resistance Mediated by Bacterial Enzymes

**Figure 1:**
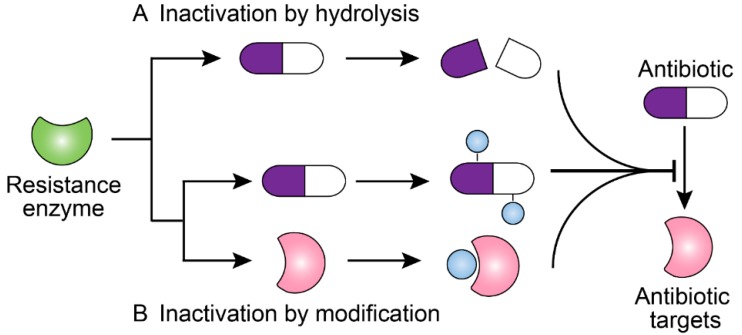
Scheme of antibiotic resistance mechanisms mediated by bacterial resistance enzymes. (**A**) Resistance enzymes hydrolyze the antibiotics and confer resistance. (**B**) Resistance enzymes modify the structure of antibiotics or antibiotic targets, preventing the antibiotics from binding to their targets and conferring resistance.

Most antibiotics act by specifically binding to their targets, thereby preventing the physiological function of these targets. However, in the long-term competition with antibiotics, some bacteria have evolved multi-detoxifying enzymes to inactivate clinically relevant antibiotics such as β-lactams, carbapenems, and aminoglycosides [42]. Based on their inactivation mechanisms, these resistance enzymes are majorly divided into hydrolytic enzymes and modifying enzymes (Figure 1).

### 2.1. Inactivation of Antibiotics by Hydrolytic Enzymes

β-Lactamases are one of the most common and important hydrolytic enzymes that inactivate penicillins, cephalosporins, carbapenems, and monobactams [43]. In particular, the evolution of β-lactam antibiotic classes with improved properties is associated with the emergence of novel β-lactamases with altered spectra of activity. For example, the early β-lactamases (penicillinase) are only active against first-generation β-lactams [44], whereas the subsequent extended-spectrum β-lactamases (ESBLs) [45] have hydrolytic activity against [46]. Alarmingly, the spread of diverse ESBLs and carbapenemases, including the imipenemase (IMP), Verona integron-encoded metallo-β-lactamase (VIM), *Klebsiella pneumoniae* carbapenemase (KPC) [47], oxacillinase (OXA) [48], and New Delhi metallo-β-lactamase (NDM) [49] enzymes in Gram-negative bacteria such as *K. pneumoniae*, *Escherichia coli*, *Pseudomonas aeruginosa,* and *Acinetobacter baumannii*, has facilitated the emergence of isolates that are resistant to all β-lactam antibiotics [50].

**Figure 2:**
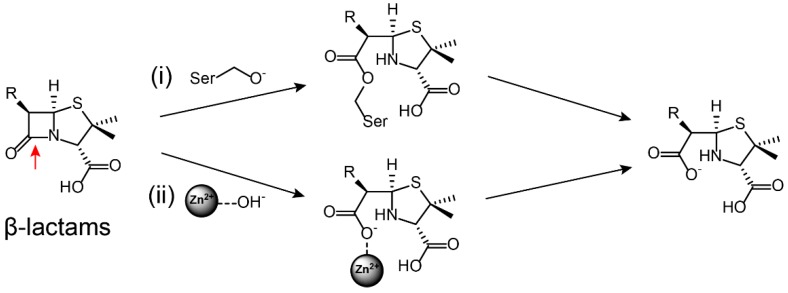
β-Lactams are hydrolized by (**i**) Ser-β-lactamase and (**ii**) metallo-β-lactamase [21]. The hydrolysis site on the antibiotic is marked by red arrows.

β-Lactamases hydrolytically cleave β-lactam antibiotics by means of two main molecular strategies: Serine residue covalently capture and destroy antibiotic activity, whereas Zn^2+^ atoms activate a water molecule to hydrolyze the antibiotics (Figure 2).

**Figure 3:**
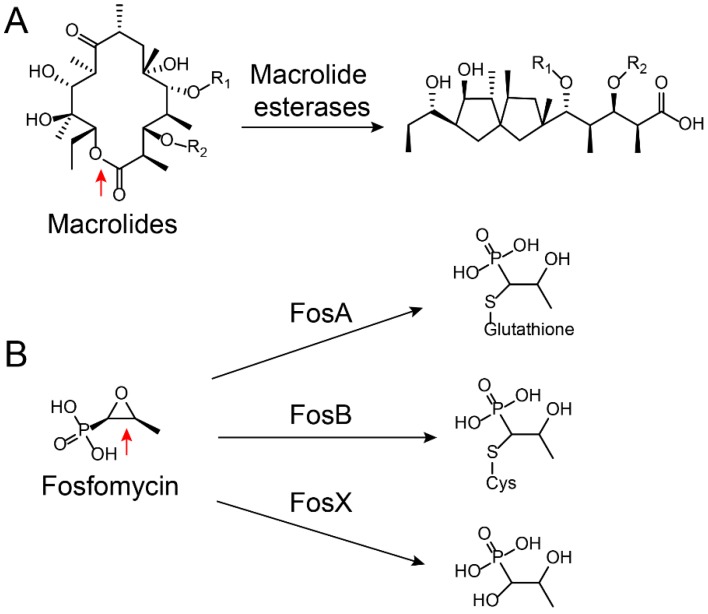
Ring-opening reactions catalyzed by (**A**) macrolide esterases [73] and (**B**) fosfomycin-resistance enzymes FosX/A/B [77]. The hydrolysis sites on the antibiotics are marked by red arrows.

Based on a classification that was first proposed by Ambler, β-lactamases are classified into four classes, namely, types A to D [51,52]. Among them, type A (penicillinase), C (cephalosporins enzyme), and D (oxacillinase) belong to Ser-β-lactamases, whereas type B refers to Zn^2+^-dependent metallo β-lactamases [53,54]. As a result of the increasing number of bacteria carrying ESBL genes, the clinical use of carbapenem antibiotics has significantly increased over the past decade. In turn, this has been accompanied by increasing clinical isolates carrying Zn^2+^-dependent metallo (type B)-β-lactamases [55,56], particularly for NDM. Since its first description in India in 2009 [57], NDM carbapenemase has become one of the most widespread carbapenemases [58,59] and it is found worldwide in numerous Gram-negative pathogens, including *A. baumannii*, *E. coli*, and *K. pneumoniae* [60,61]. Worryingly, NDM-1 and its numerous variants [62,63,64,65,66,67,68,69] confer resistance to all β-lactams except for monocyclic aztreonam [70]. Such hydrolysis also occurs for other classes of antibiotics such as macrolides and fosfomycin. Macrolide antibiotics such as erythromycin kill bacteria by binding to the large subunit of ribosomes and interfering with protein synthesis [71]. Macrolides are cyclized via an ester bond, which is the critical step for their activity. However, macrolide esterases could destroy the antibiotic’s cyclic structure by opening the ring, which [72] is then followed by an internal cyclization and dehydration through intramolecular condensation (Figure 3A) [73,74]. As a result, the ring-opened macrolides lose their antibacterial activity. Fosfomycin [75], an epoxide antibiotic, targets the enzyme UDP-*N*-acetylglucosamine enolpyruvyl transferase (MurA) that is essential for the biosynthesis of peptidoglycan [76]. Enzymatic hydrolysis to fosfomycin at position 1 occurs through the destruction of the reactive epoxide via either a thiol-containing co-substrate or a water-mediated ring opening, which depend on three similar but mechanistically distinct fosfomycin-resistance proteins, i.e., FosA, FosB, and FosX (Figure 3B) [77]. FosA, purified from Gram-negative bacteria, is a Mn^2+^- and K^+^-dependent glutathione transferase. FosB is a Mg^2+^-dependent l-cysteine thioltransferase, whereas FosX is a Mn^2+^-dependent fosfomycin-specific epoxide hydrolase [78].

### 2.2. Inactivation of Antibiotic by Modifying Enzymes

#### 2.2.1. Modification on Antibiotics

**Figure 4:**
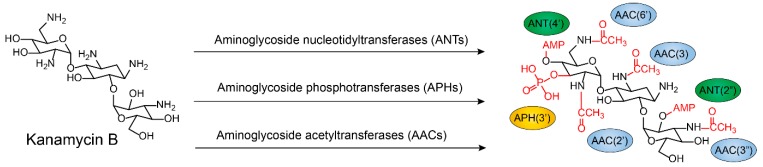
Modification of kanamycin B by aminoglycoside nucleotidyltransferases (ANTs), phosphotransferases (APHs), and acetyltransferases (AACs) [87,88,89,90,91].

Antibiotic-modifying enzymes (also termed group transferases) are the most diverse and largest family of bacterial resistance enzymes, which inactivate antibiotics by preventing binding to their respective targets. This inactivation of antibiotics depends on the modifying enzymes’ function, i.e., the addition of chemical groups to vulnerable sites of the antibiotic molecule or antibiotic targets. Various different chemical groups can be transferred, including acyl [79,80], phosphate [81], nucleotidyl [82,83], ribitoyl [42], glycosyl [84], and thiol groups [75,85,86] (Figure 4).

**Figure 5:**
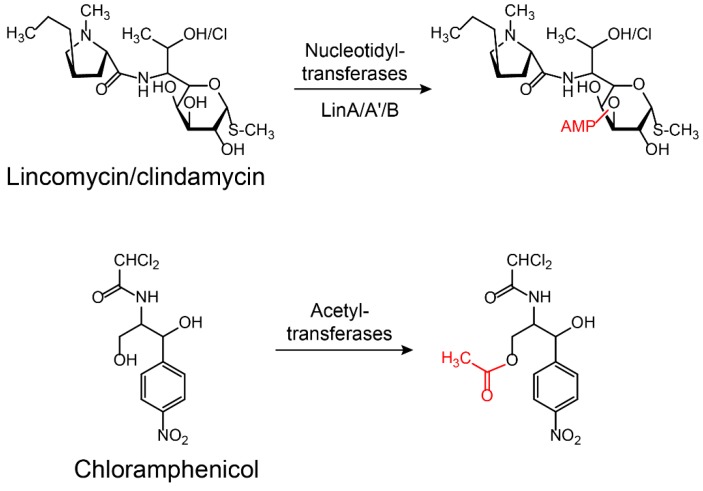
Inactivation of lincomycin/clindamycin and chloramphenicol by nucleotidyltransferases or acetyltransferases. LinA/A’/B: Lincomycin nucleotidyltransferases A/A’/B.

**Figure 6:**
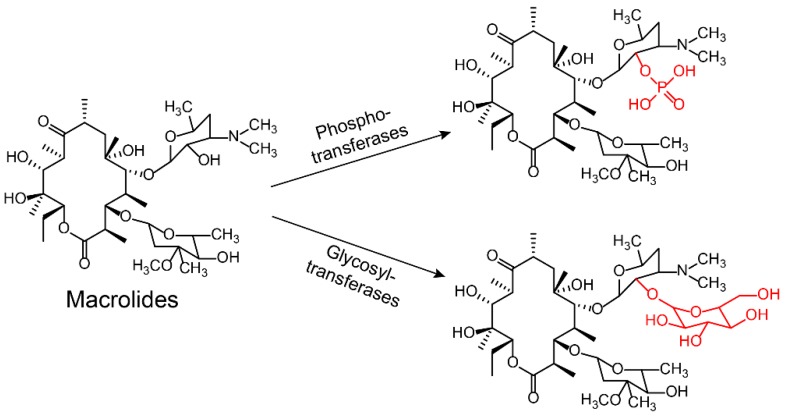
Enzyme-catalyzed inactivation of macrolides by phosphotransferases and glycosyltransferases.

**Figure 7:**
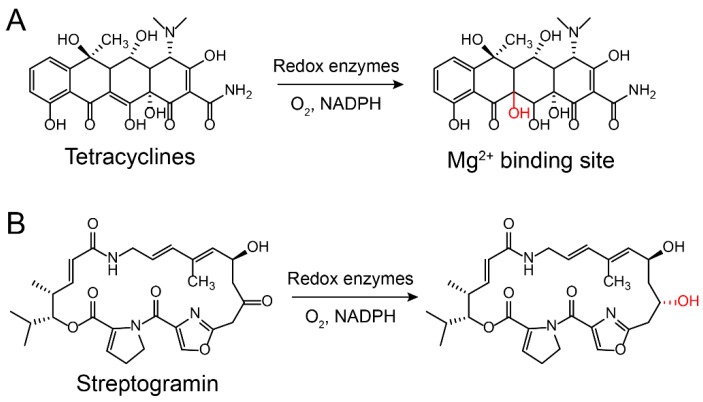
Inactivation of (**A**) tetracycline and (**B**) streptogramin by redox enzymes [95,96,97].

Aminoglycoside antibiotics are particularly vulnerable to modification as they tend to be large molecules with many exposed hydroxyl and amide groups [87]. Aminoglycoside-modifying enzymes confer high levels of resistance to antibiotics via group modification [88]. There are three principal classes of aminoglycoside-modifying enzymes: nucleotidyltransferases (ANTs), phosphotransferases (APHs), and acetyltransferases (AACs) [89,90,91]. These transferases are evolutionarily diverse and vary in the aminoglycosides that they can modify and in the part of the molecule that is modified. In addition, several other antibiotic classes are susceptible to modification, including lincomycin [92], chlormamphenicol [93] (Figure 5), and macrolides [29] (Figure 6). Except for these groups, hydroxylation mediated by redox enzymes could also confer antibiotics resistance [94]. For example, the hydroxylation of tetracycline antibiotics at position 11, which is required for antibacterial activity, disrupts the Mg^2+^-binding site of the antibiotic [95,96] (Figure 7A). Another example of redox-mediated resistance regards the antibiotic streptogramin, in which the key ketone group at position 16 is turned to hydroxymethyl, thereby making the drug inactive [97] (Figure 7B).

#### 2.2.2. Modification of Antibiotic Targets

**Figure 8:**
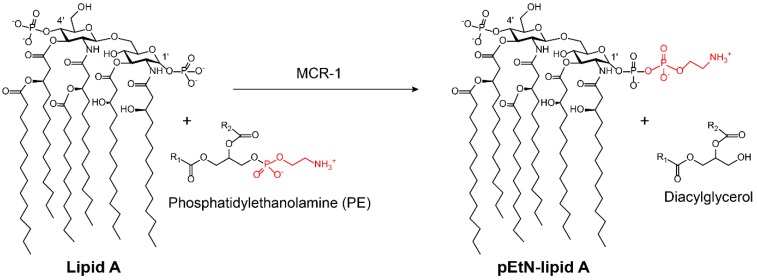
Scheme of bacterial lipid A modified by phosphatidylethanolamine (PE) through MCR-1 [121]. The modified groups on the antibiotic are in red; pEtN: phosphoethanolamine.

The vast majority of modifying enzymes inactivate antibiotics by transferring chemical groups on antibiotics’ molecules. However, there is a growing concern that bacteria can also modify antibiotics’ targets to inactivate the antibiotics. A recent example is the plasmid-borne colistin resistance mediated by MCR-1 [39]. Colistin was discovered in the 1950s [98,99] and widely used for bacterial infection control from the late 1950s to the late 1970s. This cationic peptide antibiotic was then gradually withdrawn from clinical practice owing to its potential neurotoxicity [100,101] and nephrotoxicity [102]. However, the widespread emergence of carbapenem-resistant Enterobacteriaceae (CRE) led to the re-emergence of colistin [103,104], which is currently recognized as a last-resort antibiotic against multidrug resistant Gram-negative bacteria. Colistin acts by interfering with anionic lipopolysaccharide (LPS) present in Gram-negative bacterial outer membrane [105]. However, the overuse and misuse of colistin in the clinical setting and food-producing animals have led to the emergence of colistin resistance [106]. In 2015, the first plasmid-mediated colistin resistant gene *mcr*-*1* in Enterobacteriaceae was reported in China [39]. The phosphoethanolamine (pEtN) transferase coded by *mcr*-*1* catalyzes the addition of the pEtN moiety to the phosphate groups in the lipid A component of LPS, resulting in decreased affinity between colistin and LPS (Figure 8) [39]. Most notably, the high transferability of *mcr*-*1* led to its global distribution in Gram-negative bacteria [107]. Meanwhile, a series of *mcr*-*1* variants (*mcr*-*2/3/4/5/6/7/8*) has been reported indicative of genetic adaptability [108,109,110,111,112,113,114]. Another example is the wide dissemination of plasmid-encoded chloramphenicol-florfenicol resistance (cfr) methyltransferase, which specifically methylates the adenine of position 2503 in the 23S rRNA [115,116]. Such an enzyme thereby confers resistance to a wide range of ribosome-targeted antibiotics, including phenicols, pleuromutilins, streptogramins, lincosamides, selected 16-membered macrolides, and also oxazolidinones (such as linezolid) [117,118]. Notably, cfr methyltransferase has been reported in Gram-positive and Gram-negative pathogens of both human and animal origins [119,120].

## 3. Bacterial Resistance Enzymes Inhibitors

An alternative strategy to counter the antibiotic resistance mediated by resistance enzyme is the use of inhibitors that specifically bind to the active site of enzymes and restore the activity of antibiotics. During the past decades, many enzyme inhibitors have been identified and introduced into the clinical setting, particularly for β-lactamases. In the following, we will briefly summarize the development of hydrolytic enzyme inhibitors and modifying enzyme inhibitors occurred in the past decades and highlight the novel, recently identified enzyme inhibitors.

### 3.1. Hydrolytic Enzymes Inhibitors

#### 3.1.1. Ser-β-lactamases Inhibitors

**Figure 9:**
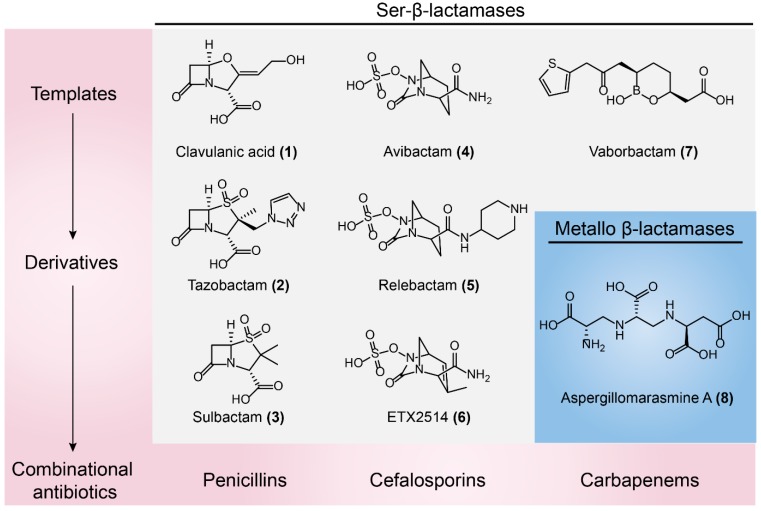
Inhibitors of Ser-β-lactamases and metallo-β-lactamases. The chemical structures, templates, derivatives of β-lactamases inhibitors, and commonly paired antibiotics are shown.

Clavulanic acid (Figure 9-(**1**)), a β-lactam-containing natural product, was the first identified β-lactamases inhibitor in the 1970s, isolated from a β-lactam antibiotic-producing strain of *Streptomyces clavuligerus* [41,122]. Clavulanic acid has poor antibiotic activity but it does show potent and irreversible inactivation of Ser-β-lactamases [123]. This unprecedented discovery gave rise to the first antibiotic/adjuvant combination that could be successfully approved in clinical use. For example, Augmentin (amoxicillin/clavulanic acid) has been applied in the clinic for over 30 years [124]. The wide use of clavulanic acid facilitated the development of other β-lactamase inhibitors, including the β-lactam sulfones tazobactam (Figure 9-(**2**)) and sulbactam (Figure 9-(**3**)) that are paired with piperacillin and ampicillin, respectively [125,126]. However, these β-lactam sulfones inhibitors are predominantly restricted to type A Ser-β-lactamases. The increase in type C and type D β-lactamases such as plasmid-borne AmpC [127] and oxacillinases [128] have progressively eroded the efficacy of these three inhibitors. To counter this threat, a new class of non-β-lactam β-lactamase inhibitor, diazabicyclooctanes (DABCOs), was introduced into clinical practice [129]. For example, avibactam (Figure 9-(**4**)) maintains the capacity to covalently acylate its β-lactamase targets [130], including CTX-M [131,132,133], AmpCs, and *K. pneumoniae* carbapenemase (KPC) [134,135]. Avycaz (a combination of ceftazidime and acibactam) was approved for clinical use by the FDA in 2015 [136]. Besides, DABCO relebactam (Figure 9-(**5**)) paired with imipenem or cilastatin are also being investigated [137,138]. Notably, neither avibactam nor relebactam are effective against *A. baumannii* infections, owing to a wide variety of class D β-lactamases expressed by this micoorganism [139]. By modifying DABCO scaffold to extend its spectrum of activity to a broad range of class D enzymes, ETX2514 (Figure 9-(**6**)) was optimized as the best candidate with high inhibition activity towards clinically relevant class A, C, and D β-lactamases and penicillin-binding proteins (PBPs). Importantly, the combination of sulbactam and ETX2514 shows high efficacy in MDR *A. baumannii* mouse infection models [140]. In addition, a novel structural β-lactamases inhibitor, Vaborbactam (Figure 9-(**7**)) based on a cyclic boronic acid pharmacophore was discovered, which is used paired with meropenem against Gram-negative bacteria, particularly for KPC-producing carbapenem-resistant Enterobacteriaceae (CRE) [141] (Figure 9).

#### 3.1.2. Metallo-β-lactamases Inhibitors

**Figure 10:**
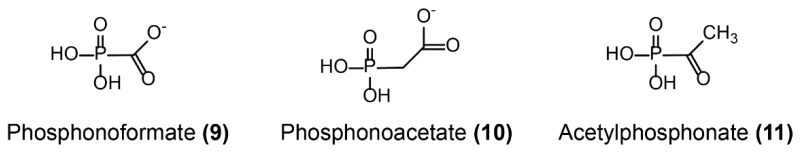
Inhibitors of the fosfomycin-resistance enzyme FosA.

Except for Ser-β-lactamase inhibitors, many efforts are being made to identify potential metallo-β-lactamases inhibitors because of the increasing concern for MDR Gram-negative bacterial infections. There are two different strategies to identify clinically useful metallo-β-lactamases inhibitors, namely, Zn^2+^-dependent and Zn^2+^-independent strategies. Because Zn^2+^ ions are essential for enzymes catalysis, most studies have focused on zinc ion chelation. For example, the well-known blood pressure-modulating drug l-captopril and its d-stereoisomer have proven to be useful inhibitors of several metallo-β-lactamases enzymes, including NDM-1, VIM-1, and IMP-7 in vitro [142,143]. Besides, some previously approved thiol-containing drugs and their derivatives, including thiorphan, tiopronin, and dimercaprol, inhibit the activity of NDM-1, VIM-1, and IMP-7 [144]. By means of an NMR counter-screening of selected compounds, Schofield’s group found that isoquinoline showed affinity for VIM-2 but not for other enzymes. Crystallographic analysis revealed that the exocyclic carboxylate displays stronger hydrogen-bonding interactions with the conserved Arg228 and Asn233 of the enzymes [145]. Notably, this is the first report about non-metal ligand inhibitors of metallo-β-lactamases, providing a new avenue for inhibitors screening. Nevertheless, a few effective inhibitors both in vitro and in vivo were identified [146]. Aspergillomarasmine A (Figure 9-(**8**)), a fungus-derived natural product [147,148] discovered by a cell-based screen for inhibitors of NDM-1, fully restored the activity of meropenem in mice infection models expressing NDM-1 *K. pneumoniae* [149]. Besides, several inhibitors were also discovered that target other hydrolytic enzymes. For instance, several simple phosphonates, including the antiviral compound phosphonoformate (*K*_i_ = 0.4 ± 0.1 µM, *K*_d_ ≈ 0.2 µM), phosphonoacetate, and acetylphosphonate (Figure 10-(**9/10/11**)), were shown to be potent inhibitors of the fosfomycin-resistance protein FosA [150].

### 3.2. Modifying Enzymes Inhibitors

#### 3.2.1. Aminoglycoside Modifying Enzymes Inhibitors

**Figure 11:**
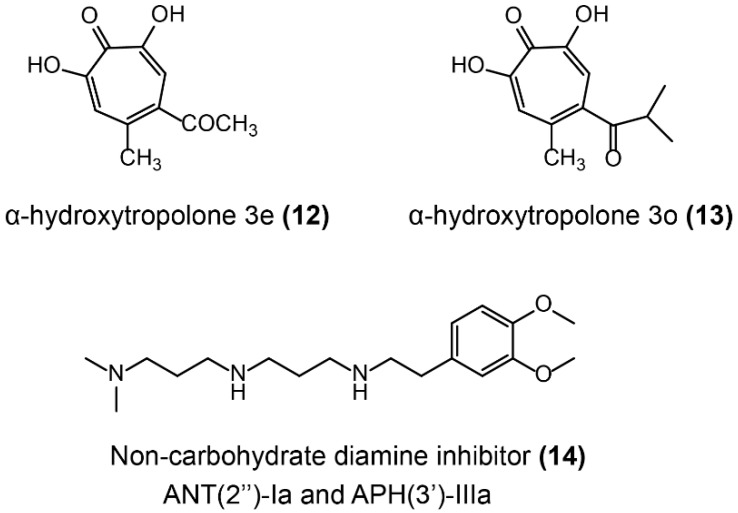
Inhibitors of aminoglycoside ANTs.

**Figure 12:**
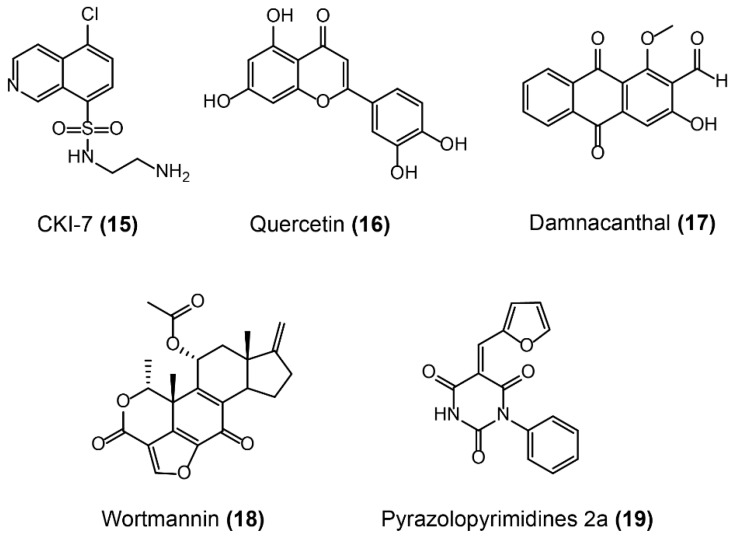
Inhibitors of APHs.

**Figure 13:**
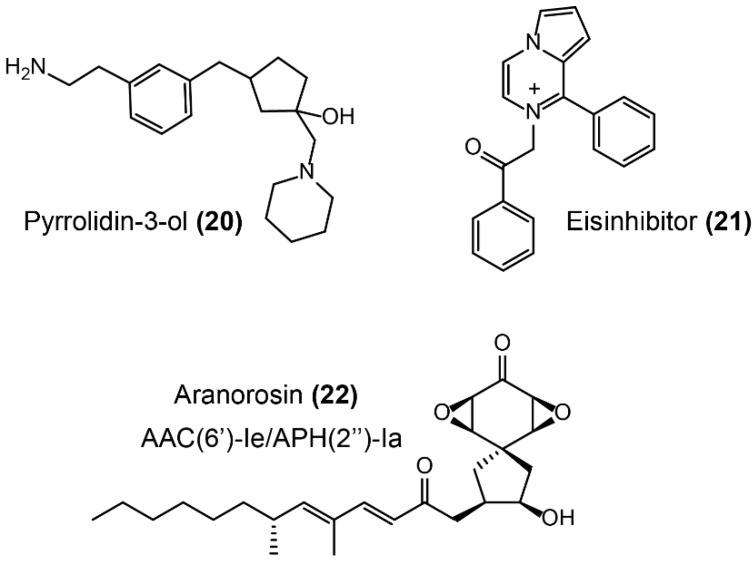
Inhibitors of AACs.

**Figure 14:**
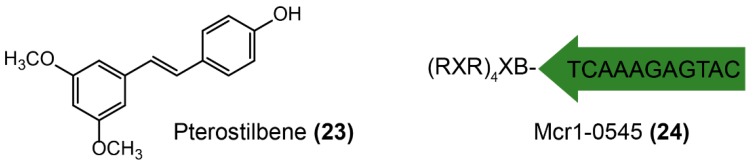
Inhibition of MCR-1 by molecules directly interacting with resistance enzymes or by targeting its mRNA.

Aminoglycoside antibiotics are the foremost bactericidal drugs that specifically target the ribosome [151], which show potent antibacterial activity against both Gram-negative and Gram-positive pathogens. For instance, gentamicin, tobramycin, and amikacin are still widely used in clinical settings [152]. The prevalent resistance mechanisms of aminoglycoside antibiotics have been briefly described in Section 2.2, especially the aminoglycoside-modifying enzymes (AMEs) including nucleotidyltransferases (ANTs), phosphotransferases (APHs), and acetyltransferases (AACs). Some AMEs inhibitors have been reported to inhibit ANTs or multiple enzymes and restore antibiotics’ activity against resistant bacteria (Figure 11). The oxidopyrylium cycloaddition/ring-opening strategy was applied to determine the inhibition activity of a library of synthetic a-hydroxytropolones against ANT(2″)-Ia. As a consequence, two of these synthetic compounds, including α-hydroxytropolones 3e and 3o (Figure 11-(**12/13**)), restored gentamicin activity against ANT-(2″)-Ia-expressing bacteria [153]. Besides, some compounds showed evidence that they simultaneously inhibit multiple AMEs. A library of 45 non-carbohydrate AME inhibitors (Figure 11-(**14**)) was designed based on the 1,3-diamine pharmacophore found in aminoglycoside structures and were shown to be competitive inhibitors of ANT(2″)-Ia and APH(3′)-IIIa [154]. A detailed structure–activity relationship study of non-carbohydrate inhibitors revealed that the 3-(dimethylamino) propylamine moiety was critical for their inhibitory activity [155].

Similarly, several potent inhibitors of APHs were identified (Figure 12). Because of the high similarity of APHs to eukaryotic protein kinases in both protein structure and mechanism, protein kinase inhibitors were investigated as potential inhibitors of APHs, including CKI-7 [156], the aforementioned natural product kinase inhibitor quercetin [157], and damnacanthal (Figure 12-(**15/16/17**)) [158]. Besides, the lipid PI-3 kinase inhibitor wortmannin (Figure 12-(**18**)) also displays inhibitory activity towards APH(2´´)-Ia [159], owing to the analogous protein conformation between lipid kinases and APHs. However, these inhibitors may nonspecifically bind to eukaryotic protein kinases or lipid kinases in vivo, resulting in undesirable side effects. Therefore, compounds with a highly selective binding mechanism to AMEs would have a beneficial outcome, particularly for their safety in vivo. Meaningfully, pyrazolopyrimidines such as 2a (Figure 12-(**19**)) binds to and inhibits APH(3´)-I (a dominant resistance mechanism in Gram-negative pathogens) by a pathway that is distinct from the binding mode of human kinases [160], offering a potential route for derivatives that selectively inhibit APHs over human targets.

The development of computer-assisted technology undoubtedly facilitates a versatile screening approach for novel resistance enzyme inhibitors, particularly for high-throughput screening (HTS). For instance, in silico molecular docking simulation provides a viable strategy to identify enzyme inhibitors [161,162,163]. One classic example is Glide, which offers a rapid, accurate docking and scoring approach for the screening of potential inhibitors [164]. Unlike other methods for docking ligands to the rigid 3D structure of a known protein receptor, Glide approximates a complete systematic search of the orientation, conformation, and positional space of the docked ligand [165,166]. These characters confirm its higher accuracy with respect to other methods such as GOLD [167,168], FlexX [169,170], and Surflex [171,172]. By employing this strategy to screen a collection of 280,000 compounds, 1-[3-(2-aminoethyl)benzyl]-3-(piperidin-1-ylmethyl)pyrrolidin-3-ol (Figure 13-(**20**)) was found. It inhibits the acetylation of aminoglycosides in vitro with IC_50_ values of 39.7 and 34.9 μM using kanamycin A or amikacin as substrates [173]. To screen the inhibitors of AACs from *Mycobacterium tuberculosis*, Keith et al. tested 23,000 molecules, of which 300 (1.3%) showed a reasonable degree of inhibition against AACs, and one compound (Figure 13-(**21**)) showed IC_50_ values of 0.364 μM [174]. Aranorosin (Figure 13-(**22**)), a natural product isolated from *Gymnascella aurantiaca*, circumvents aminoglycoside antibiotics resistance by inhibiting AME AAC(6′)-Ie/APH(2′′)-Ia [175] (Figure 13).

#### 3.2.2. MCR-1 Inhibitors

Two successful strategies were applied for screening MCR-1 inhibitors, including directly looking for molecules interacting with MCR-1 protein and targeting its mRNA (Figure 14). Pterostilene (Figure 14-(**23**), trans-3,5-dimethoxy-4′-hydroxystilbene), isolated from fresh leaves or fruit, has been previously reported owing to its potent anticancer [176,177], anti-inflammatory [178], and antioxidant activities [179]. Recently, pterostilene was found to be a novel MCR-1 inhibitor [180], which rescues the efficacy of polymyxin B both in vitro and in vivo. A significant synergistic effect between pterostilbene and polymyxin B (fractional inhibitory concentration index 0.156 or 0.188) against MCR-producing *E. coli* of both human and animal origins was observed. However, more mechanistic studies are required to elucidate the potentiation activity of pterostilene. Transcription inhibition of enzyme-coding genes also provides a potential approach to rescue antibiotic activity, such as specifically designed antisense oligonucleotides [181,182,183], which suppress bacterial genes expression on the basis of complementary interaction. The obstacle that mostly limits the adjuvant potential of antisense oligonucleotides is the highly impermeable bacterial membrane [184]. Besides, bacteria do not initiatively uptake oligonucleotides from their environment [185]. Therefore, conjugation with vectors such as lipids [186], cell-penetrating peptides [187], polymers [188], and nanoparticles [189] would facilitate the delivery of antisense oligonucleotides to various bacterial cells. For example, an optimized cationic peptide-conjugated phosphorodiamidate morpholino oligomer (PPMO) Mcr1-0545 (Figure 14-(**24**)) was designed to target *mcr*-*1* mRNA, preventing translation and modulating colistin resistance [190]. Excitingly, the combination of Mcr1-0545 with colistin offered a significant survival advantage in the murine sepsis infection model. Meaningfully, such antisense RNA strategies could be expanded to other applications for combating antibiotic bacteria by targeting different function genes [191,192,193], including bacterial essential gene (thus killing bacteria, like antibiotics) [194,195,196,197], resistance genes (thus rescuing antibiotic activity, like adjuvants) [190,198], and virulence genes (thus preventing bacterial pathogenesis, like anti-virulence agents) [199,200,201,202].

## 4. Outlook and Conclusions

Although numerous compounds that inhibit different bacterial resistance enzymes have been found, instances of their clinical use are limited to β-lactamases inhibitors. There are two major concerns, that is, low efficacy and unspecific toxicity of these compounds in vivo. It is noteworthy that some candidates failed to demonstrate significant efficacy in vivo due to their liability to degradation by proteases. Thus, activity screening in vitro alone is not enough to show evidence of their therapeutic potential. Alternatively, some animal infection models could be straightforwardly utilized to evaluate the therapeutic effectiveness of identified leads, such as the silkworm infection model, which has conserved immune response and comparable pharmacokinetics with mammals [203]. In addition, the toxicity of some enzymes inhibitors is also a key factor that leads to failure during safety evaluation. This problem could be addressed to identify more specific resistance enzyme inhibitors, which specifically bind to prokaryotic (but not eukaryotic) enzymes. Such a solution requires a better understanding of the conformation of resistance enzymes and of the binding sites of the inhibitors.

Antibiotics resistance, caused by bacterial resistance enzymes that have hydrolytic and modifying effects, poses a foremost and serious threat to public health worldwide. Despite the challenges faced in the screening of novel resistance enzyme inhibitors and in bringing them into the market, the discovery of enzyme inhibitors provides a complementary strategy to new antibiotic discovery. Importantly, these inhibitors can potentiate or restore the antibacterial activity of some promising antibiotics that are vulnerable and can be hydrolyzed or modified. The wide application of β-lactamase inhibitors in the clinic over the past decades is the most robust proof of concept. In addition, the pairing of inhibitors with antibiotics also contributes to minimizing the burden and emergence of antibiotic resistance. Overall, resistance enzyme inhibitors show great promise. Nevertheless, other novel strategies should be continuously explored to preserve the efficacy of antibiotics in this resistance era.
